# Factors Associated with Promoted Proliferation of Osteosarcoma by Peptidylarginine Deiminase 4

**DOI:** 10.1155/2021/5596014

**Published:** 2021-05-15

**Authors:** Jianping Guo, Lei Yin, Xuezhong Zhang, Peng Su, Qiaoli Zhai

**Affiliations:** ^1^Department of Oncology, Maternal and Child Health Care Hospital of Zibo, Zibo 255029, China; ^2^Human Resources Department, Zibo Central Hospital, Zibo 255036, China; ^3^Department of Laboratory Medicine, Zibo Central Hospital, Zibo 255036, China; ^4^Center of Translational Medicine, Zibo Central Hospital, Zibo 255036, China

## Abstract

Osteosarcoma is the most common type of bone malignancy, and the pathogenesis has not been entirely elucidated yet. An important deimination modification enzyme PADI4 (peptidylarginine deiminase 4) has attracted much attention in recent years for its important function in several kinds of human tumors. However, the role of PADI4 on osteosarcoma tumorigenesis remains largely unrevealed. Here, we first assessed the effect of PADI4 on osteosarcoma proliferation by the CCK8 method and colony formation assay. Ectopically expressing PADI4 positively regulates the colony formation capacity of both U2OS and Saos-2 cells. Furthermore, we explored the related mechanism and showed that PADI4 could stimulate Wnt/*β*-catenin and MEK/ERK signaling in both U2OS and Saos-2 cells. Then, we detected expression of PADI4 in human tissues of osteosarcoma and revealed that differential expression of PADI4 was associated with tumorigenesis of osteosarcoma. Last, we performed the *in vivo* experiment in nude mice and results also showed PADI4 could affect the tumor growth. In conclusion, this work revealed that PADI4 could upregulate the proliferation of osteosarcoma, mainly via the Wnt/*β*-catenin and MEK/ERK signaling pathway. This study gives us new insight into the regulation mechanism of osteosarcoma proliferation and highlights PADI4 as a promising target for osteosarcoma diagnosis and treatment.

## 1. Background

As the most common type of bone malignancy, osteosarcoma frequently occurs in children and adolescence [1]. The mesenchymal cell line is its source. The formation of tumor-like bone tissue in the cartilage stage leads to the rapid growth of osteosarcoma [2]. Improving the survival rate of the disease mainly depends on early diagnosis and timely treatment [3, 4]. However, due to its high mortality, the mechanism of osteosarcoma still needs to be further explored [5].

Peptidylarginine deiminase 4 (PADI4) can catalyze the deimination of arginine into citrulline, regulate the expression of target genes, and participate in a variety of cellular processes [6]. It has received extensive attention in recent years, especially in immune response and tumor development [7, 8]. It has been reported that PADI4 is involved in the tumorigenesis of several tumors, such as ovarian cancer and esophageal squamous cell carcinoma [9–11]. The dominate mechanism by which PADI4 functions is to alter the chromatin structure or to change the modification of target histones or other nonhistone proteins [12–15]. However, the effect and related mechanism of PADI4 on the progression of osteosarcoma remain largely unrevealed.

Our present study has shown the upregulated expression of PADI4 in osteosarcoma tissues in comparison with the matched adjacent tissues. PADI4 could promote osteosarcoma cell proliferation both *in vitro* and in a mouse xenograft model. Additionally, we also revealed that the upregulation of osteosarcoma proliferation by PADI4 is primarily through the Wnt/*β*-catenin and MEK/ERK signaling pathway.

## 2. Materials and Methods

### 2.1. Osteosarcoma Tissue Samples

All osteosarcoma specimens and their counterpart adjacent tissues were obtained from Zibo Central Hospital, and all tissues were confirmed by pathological diagnosis. This study was performed in accordance with the guidelines of the ethics committee of Zibo Central Hospital and was carried out with the principles of the Declaration of Helsinki.

### 2.2. Cell Culture and Transfection

The human normal osteoblast cell line hFOB1.19 and the human osteosarcoma cell lines U2OS, Saos-2, HOS, 143B, and SJSA1 were obtained from the Type Culture Collection of the Chinese Academy of Sciences (Shanghai, China). The medium and FBS used to culture cells were purchased from Gibco (USA). The 1640 medium was applied to culture U2OS cells supplemented with 10% FBS. McCoy's 5A medium was applied to culture Saos-2 cells supplemented with 10% FBS. HOS, 143B, and SJSA were cultured in DMEM/F12 medium supplemented with 10% FBS at 37°C with 5% CO_2_. hFOB1.19 was routinely cultured with rapid cell division in DMEM/F12 supplemented with 10% FBS at 33.5°C with 5% CO_2_.

The PADI4 overexpression and knockdown constructs used in this work were kindly gifted from the Department of Biochemistry and Molecular Biology, Peking Union Medical College, China. Cells were transfected with these constructs using Lipofectamine 3000 (Invitrogen) according to the manufacturer's protocol.

### 2.3. CCK8 Method

Cell Counting Kit-8 (CCK-8; Dojindo, Tokyo, Japan) was used to detect the proliferation of osteosarcoma cells according to the manufacturer's protocol. The cell seeding density is 1 × 10^3^ cells in each well of a 96-well plate, and the test is performed every 24 hours for a total of 5 days. Then, add the CCK8 solution and incubate the cells at 37°C for 1 to 2 hours. Measure the absorbance using a microplate reader (Thermo Fisher, USA) at 450 nm.

### 2.4. Western Blotting

Prepare whole cell lysate with RIPA buffer from cultured cells. Separate by SDS-PAGE and transfer to a polyvinylidene fluoride membrane, then block the protein on the membrane with 5% skim milk for 1 hour, and then, combine with the primary antibody (anti-PADI4 1 : 1000, anti-ERK 1 : 1000, antiphospho ERK 1 : 1000, anti-MEK 1 : 1000, antiphospho MEK 1 : 1000, anti-*β*-catenin 1 : 1000, antiactive *β*-catenin 1 : 1000, anti-H3-Cit 1 : 1000, and anti-GAPDH 1 : 3000; antibodies purchased from CST, USA) overnight at 4°C. The membrane was washed with TBST, then incubated with HRP-conjugated secondary antibody at the concentration of 1 : 5000 for 1 hour at RT. The Tanon 5200 chemiluminescence image analysis system (Tanon, China) was used to visualize chemiluminescence.

### 2.5. Reverse Transcription-Polymerase Chain Reaction (RT-PCR)

Use TRIzol (Invitrogen) to extract total RNA from cells, and then, use a cDNA Synthesis Kit (TaKaRa) for reverse transcription. For PCR amplification, a kit of SYBR Premix Ex Taq II purchased from TaKaRa was applied. Sequences for primers used in this work are listed as follows: PADI4-forward: CCCAAACAGGGGGTATCAGT; PADI4-reverse: CCACGGACAGCCAGTCAGAA. AXIN2-forward: GAAACAGCTCCAGAGAGAAATG; AXIN2-reverse: GCTCTCCAACTCCAGCTTCAG. CCND1-forward: GAGGAGAACAAACAGATCATCC; CCND1-reverse: GGTAGTAGGACAGGAAGTTGTT. DKK1-forward: CACTGATGAGTACTGCGCTAGT; DKK1-reverse: TCAGAAGACACACATATTCCAT. MYC-forward: ACCACCAGCAGCGACTCTGAGG; MYC-reverse: TCCAGCAGAAGGTGATCCAGAC. GAPDH-forward: GAAGGTGAAGGTCGGAGTC; GAPDH-reverse: GAAGATGGTGATGGGATTT.

### 2.6. Colony Formation Assay

Cells were seeded in 6-well plates at a density of 1 × 10^3^ per well and cultured for about 10 days when visible colonies began to form. The cells were fixed with 4% paraformaldehyde after washing with PBS for 3 times. Then, the cells were stained with 0.1% crystal violet for 20 min, and the number of colony formations was counted and photographed.

### 2.7. Immunohistochemistry Staining

Immunohistochemistry staining was performed according to the manufacturer instruction of the kit (Zhongshanjinqiao, Beijing, China). In detail, paraffin sections were deparaffinized in xylene and rehydrated through graded alcohol. After antigen enzymatic retrieval, sections were incubated with 3% hydrogen peroxide to block the activity of endogenous tissue peroxidase. After being blocked with 1% bovine serum albumin, sections were incubated with PADI4, p-ERK, or active *β*-catenin antibodies (CST, Beverly, USA) at 4°C overnight. Sections were incubated with streptavidin-peroxidase conjugate after secondary antibody incubation at 37°C for 25 min, and then, DAB staining was performed. Finally, sections were gently counterstained with hematoxylin and dehydrated through graded alcohol. Staining was observed with a microscope (Olympus, Tokyo, Japan). The integrated optical density (IOD) of PADI4 and E-cadherin staining was quantitatively analyzed using Image-Pro Plus 6.0 software.

### 2.8. Animal Models

Approximately 1 × 10^7^ cells containing the control vector or the PADI4 lentiviral vector were subcutaneously injected into 6-week-old female nude mice which were purchased from Shanghai SLAC Laboratory Animal Co. Ltd. (Shanghai, China). Measure and calculate the tumor volumes every 7 days. 35 days after injection, mice were killed ultimately, and the tumor tissues were collected for immunohistochemistry staining. The protocol was approved by the Animal Care and Use Committee of Zibo Central Hospital.

### 2.9. Statistical Analysis

GraphPad Prism was used to perform statistical analysis. Results were presented as mean ± standard deviation (SD). The statistical significance was analyzed by Student's *t* test, ANOVA, or chi-square test. *P* < 0.05 indicated significant difference statistically.

## 3. Results

### 3.1. The Upregulation of Proliferation of Osteosarcoma Cells by PADI4

**Figure 1:**
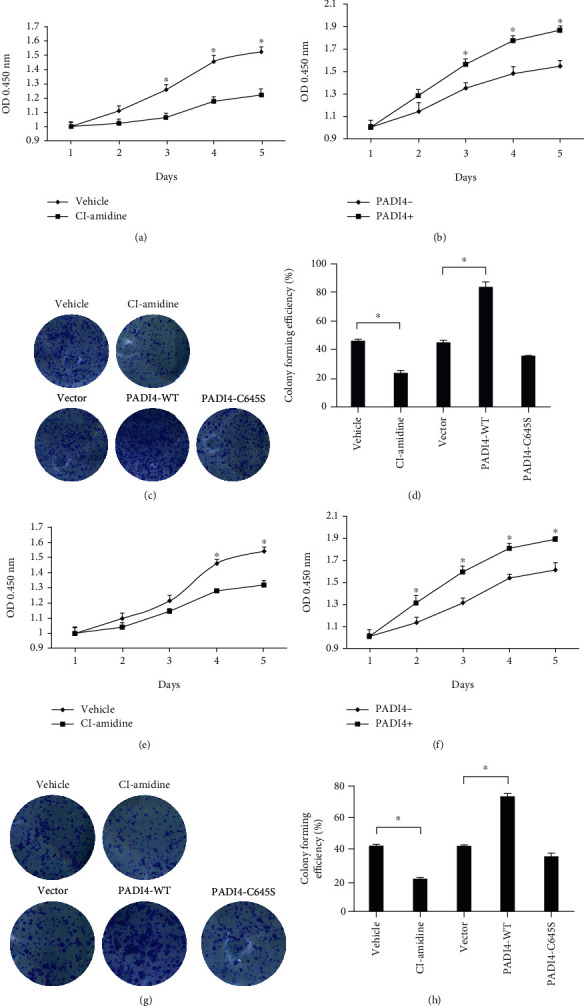
PADI4 upregulates proliferation and colony formation of U2OS and Saos-2 cells. (a, e) U2OS cells (a) and Saos-2 cells (e) were treated with PADI4 inhibitor Cl-amidine, and cell viability was detected by CCK8 assay. (b, f) Cell viability was detected by CCK8 assay after overexpression of PADI4 in U2OS (b) and Saos-2 cells (f). (c, g) The colony forming capacity was measured in U2OS (c) and Saos-2 cells (g) after treatment with Cl-amidine or being transfected with PADI4 constructs. (d, h) Analyze and calculate the colony density by ImageJ. Data are presented as the mean ± SEM of three independent experiments (^∗^*P* < 0.05).

U2OS and Saos-2 cells were treated with PADI4 inhibitor Cl-amidine as well as ectopically expressing PADI4, and CCK8 assay was performed to investigate cell growth. Results indicated that Cl-amidine could remarkedly suppress the proliferation of U2OS and Saos-2 cells (Figures 1(a) and 1(e)) while overexpressing PADI4 significantly promoted cell proliferation (Figures 1(b) and 1(f)). Colony formation was next performed to further detect the effect of PADI4 on the proliferation of U2OS and Saos-2 cells. The results showed that colony formation capacity of the PADI4 inhibitor treatment group was significantly lower than that of the control group. Overexpression of PADI4 remarkedly promoted the colony formation capacity of U2OS and Saos-2 cells. PADI4-C645S, which has lost deimination activity, showed less effect than PADI4-WT (Figures 1(c) and 1(d) and 1(g) and 1(f)). These results revealed that PADI4 could upregulate the proliferation ability of osteosarcoma cells *in vitro*.

### 3.2. PADI4 Promoted the Osteosarcoma Cell Proliferation via Upregulation of Wnt/*β*-Catenin and MEK/ERK Signaling

**Figure 2:**
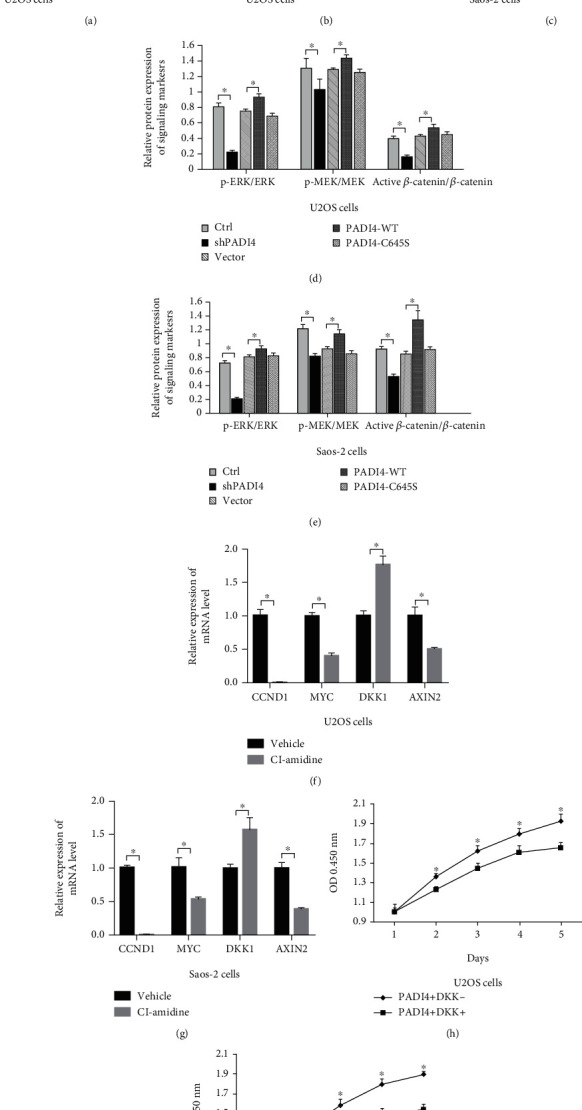
The effect of PADI4 on the markers of Wnt/*β*-catenin and MEK/ERK signaling. (a) Expressions of Wnt/*β*-catenin and MEK/ERK signaling markers were detected by Western blot after treatment with U2OS cells with Cl-amidine for the indicated time. (b, c) Protein levels of markers involved in Wnt/*β*-catenin and MEK/ERK signaling were detected by Western blot after PADI4 knockdown or overexpression in both U2OS (b) and Saos-2 (c) cells. (d, e) Quantitative analysis of protein expression levels for markers demonstrated in both U2OS (d) and Saos-2 (e) cells. (f, g) mRNA expression of markers involved in Wnt/*β*-catenin signaling was analyzed by RT-PCR after Cl-amidine treatment in both U2OS (f) and Saos-2 (g) cells. (h, i) Cell viability detection was performed by CCK8 assay after transfecting PADI4 with or without DKK1 plasmids for the indicated time in both U2OS cells (h) and Saos-2 cells (i).

As Wnt/*β*-catenin and MEK/ERK signaling was involved in the tumorigenesis of several human tumors and bone development, next, we assessed the effect of PADI4 on the activity of Wnt/*β*-catenin and MEK/ERK signaling in osteosarcoma cells. The results in Figure 2(a) showed that active markers in these signaling types, such as phosphorylation of MEK and ERK, as well as active *β*-catenin, were downregulated after the PADI4 inhibitor treatment. We also overexpressed PADI4-WT/C645S and PADI4 shRNA to detect the impact on Wnt/*β*-catenin and MEK/ERK signaling. Results showed that knockdown of PADI4 significantly suppressed phosphor-MEK, phosphor-ERK, and the active form of *β*-catenin, while overexpression of wild-type PADI4 promoted these markers. Additionally, PADI4-C645S had little effect on these markers of the two signaling types (Figures 2(b)–2(e)). These results clearly showed PADI4 could promote Wnt/*β*-catenin and MEK/ERK signaling in U2OS and Saos-2 cells.

Furthermore, in order to confirm the regulation of the PADI4 inhibitor on the mRNA level of downstream genes of *β*-catenin, we also performed qRT-PCR assay in osteosarcoma cells (Figures 2(f) and 2(g)). Results showed that the mRNA expression of CCND1 and MYC, which have been identified as the major target genes of *β*-catenin, was suppressed after Cl-amidine treatment. Significantly, the relative expression level of CCND1 was dramatically affected by PADI4 inhibition, falling to about 0.002. Expression of DKK1 regulated by PADI4 was also detected. DKK1 could upregulate the endocytosis of Wnt receptors LRP5 and LRP6 and thus suppress Wnt/*β*-catenin signaling. Results showed that Cl-amidine induced the mRNA level of DKK1. However, the expression of AXIN2 was downregulated after treatment with Cl-amidine. In order to further confirm the role of DKK1 in PADI4-mediated osteosarcoma cell proliferation, we transfected osteosarcoma cells with DKK1 plasmid together with PADI4 overexpression. The detection of cell viability using CCK8 methods clearly demonstrated that DKK1 overexpression reversed the promotion effect of PADI4 on osteosarcoma cell proliferation (Figures 2(h) and 2(i)).

### 3.3. Increased PADI4 Expression Is Positively Correlated with Osteosarcoma Progression in Clinical Tissues

**Figure 3:**
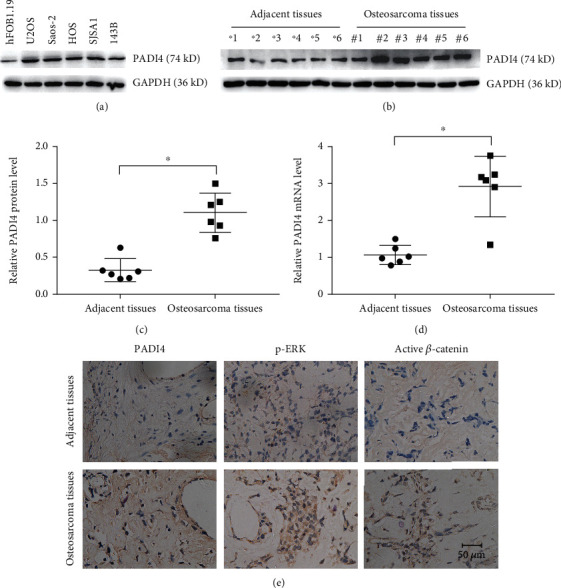
Increased expression of PADI4 in osteosarcoma tissues. (a) Expression levels of PADI4 protein in normal human osteoblast cell (hFOB1.19) and several human osteosarcoma cell lines (U2OS, Saos-2, HOS, SJSA1, and 143B) by Western blot. GAPDH was used as a loading control. (b) Western blot was performed to detect the expression levels of PADI4 in human osteosarcoma tissues and adjacent tissues. GAPDH was used as a loading control. (c) Quantification of protein expression. ^∗^*P* < 0.05 vs. adjacent tissues. (d) Expression of PADI4 mRNA in human osteosarcoma tissues and adjacent tissues was investigated by RT-PCR analysis. Data are presented as the mean ± SEM. ^∗^*P* < 0.05 vs. adjacent tissues. (e) Immunohistochemistry staining of PADI4, p-ERK, and active *β*-catenin in human osteosarcoma tissues and adjacent tissues.

Next, we performed Western blot and RT-PCR to explore the relevance of PADI4 expression in osteosarcoma samples and paired normal tissues. Firstly, we detected the protein expression of PADI4 in the normal human osteoblast cell (hFOB1.19) and several human osteosarcoma cell lines (U2OS, Saos-2, HOS, SJSA1, and 143B) by Western blot, and the results indicated increased PADI4 expression in osteosarcoma cells (Figure 3(a)). In order to investigate the role of PADI4 in osteosarcoma proliferation in clinical samples, we then analyzed the correlation between PADI4 expression and clinicopathological parameters. The results are displayed in Tables 1 and 2 and suggested that a higher expression of PADI4 was correlated with a larger tumor size. Next, we examined the mRNA and protein expression level of PADI4 in both osteosarcoma tissues and adjacent tissues. Results showed the upregulated expression of PADI4 in osteosarcoma tissues (Figures 3(b)–3(d)). We further validated the expression of PADI4 and Wnt/*β*-catenin and MEK/ERK signaling pathway proteins (p-ERK and active *β*-catenin) by means of IHC in tumor and adjacent normal tissues. Results showed increased staining of PADI4, p-ERK, and active *β*-catenin in osteosarcoma tissues compared with adjacent normal tissues (Figure 3(e)).

### 3.4. PADI4 Accelerates the Xenograft Growth of Osteosarcoma *In Vivo*

**Figure 4:**
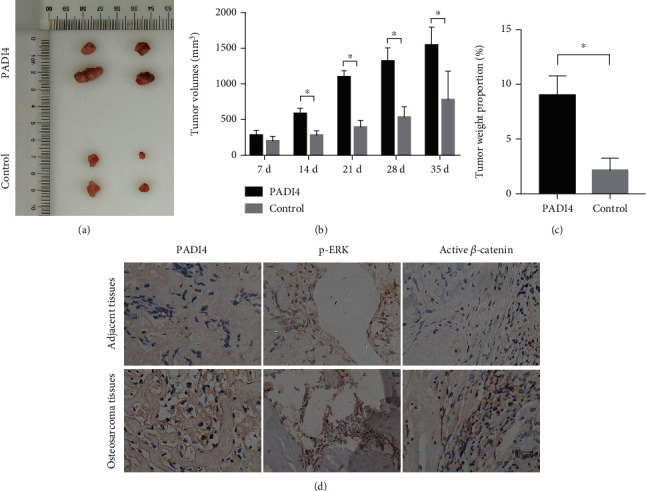
The effect of PADI4 overexpression on the xenograft growth of osteosarcoma *in vivo*. (a) Images of tumor formation after the mice were injected with cells that had been transfected with PADI4 or vector constructs. (b) Volume detection of xenograft tumor. (c) Weight proportion detection of xenograft tumor. ^∗^*P* < 0.05 indicated statistical significance. (d) 35 days after injection, mice were killed ultimately, and the tumor tissues were collected for immunohistochemistry staining of PADI4, p-ERK, and active *β*-catenin.

Tumor formation experiments in nude mice were performed to further confirm the role of PADI4 in osteosarcoma tumorigenesis *in vivo*. The tumor growth is detected after osteosarcoma xenografts, and the results showed that the growth of the transplanted tumors including its volume and weight remarkedly increased with PADI4 overexpression (Figures 4(a)–4(c)). 35 days after injection, the mice were killed ultimately, and the tumor tissues were collected for immunohistochemistry staining of PADI4, p-ERK, and active *β*-catenin. It is shown that PADI4, p-ERK, and active *β*-catenin had higher expression levels in osteosarcoma tissues than in adjacent normal tissues in these mice (Figure 4(d)).

## 4. Discussion

Peptidylarginine deiminase 4 have attracted more and more attention due to its role in tumorigenesis [9]. However, as in osteosarcoma progression, the effect of PADI4 remains largely unrevealed. In this work, we suggested that PADI4 could accelerate osteosarcoma progression, and the use of PADI4 inhibitors could effectively slow down the proliferation of osteosarcoma. Cell proliferation and colony formation experiments confirmed that PADI4 inhibitor treatment downregulated the growth and colony forming ability of U2OS and Saos-2 cells, while overexpression of PADI4 promoted cell growth and colony forming ability. In addition, the PADI4 mutant without enzyme activity did not change the colony-forming ability of osteosarcoma cells, indicating that the enzyme activity of PADI4 is essential to regulate the proliferation of osteosarcoma cells.

Then, we studied the mechanism involved in the regulation of osteosarcoma proliferation mediated by PADI4. The Wnt signaling pathway is usually activated abnormally in several types of cancers and could cooperate with other signaling pathways or antagonize them to modulate tumor proliferation [16]. The accumulation of active *β*-catenin is a sign of Wnt pathway activation [17]. MEK/ERK is also a key signal transduction pathway that regulates cell proliferation and differentiation [18]. About 30% of human cancers have abnormal activation of the MEK/ERK pathway [19, 20]. Therefore, we investigated the effect of PADI4 on the expression of key markers involved in MEK/ERK and Wnt/*β*-catenin signaling pathways. The results showed that the PADI4 inhibitor significantly reduced phosphor-MEK, phosphor-ERK, and the active form of *β*-catenin. Accordant results were also detected after PADI4 knockdown. Ectopic expression of wild-type PADI4 obviously induced MEK/ERK and Wnt/*β*-catenin signal transduction which had not been affected by PADI4 mutation. Interestingly, AXIN2, which has the role to inhibit Wnt signaling by decreasing *β*-catenin stability, showed a contradictory expression. We consulted the literature and got some reasonable explanation. AXIN2 not only functions as the negative regulator of Wnt signaling but also is the downstream target gene of Wnt signaling. So AXIN2 may also be involved in the feedback regulation of Wnt signaling. Several researches have also gotten similar results as us, such as Lu et al. [21].

Compared with normal tissues, the expression of PADI4 in osteosarcoma samples was significantly higher, which also supports our conclusions in cell experiments. In addition, the results of tumorigenesis in nude mice also showed that overexpression of PADI4 promoted tumorigenesis through activating MEK/ERK and Wnt/*β*-catenin signaling pathways *in vivo*.

## 5. Conclusion

Taken together, results of this work confirmed that PADI4 can promote osteosarcoma proliferation through Wnt/*β*-catenin and MEK/ERK signaling pathways. This work gives us new insight into the mechanism exploration of osteosarcoma proliferation and indicated that PADI4 might serve as a promising target and biomarker for osteosarcoma diagnosis and therapy.

## Figures and Tables

**Table 1:** Case description.

No.	Age	Gender	Tumor size (cm)	Location	Staging	IOD of PADI4
1	15	Male	2.5	Femur	III	0.59
2	24	Female	1.3	Tibia	II	0.51
3	14	Male	4.4	Tibia	III	0.62
4	16	Female	3.2	Femur	III	0.55
5	68	Male	2.8	Femur	II	0.61
6	72	Female	2.5	Tibia	II	0.53
7	38	Female	1.6	Femur	II	0.31
8	63	Female	2.1	Tibia	III	0.54
9	22	Male	1.7	Femur	II	0.23
10	42	Female	2.4	Tibia	I	0.38
11	61	Female	4.2	Femur	III	0.67
12	65	Male	1.8	Femur	I	0.35

**Table 2:** PADI4 expression and clinicopathologic characteristics of OS patient.

Parameter	Case	PADI4 expression		*P* value^a^
		Low (*n* = 4)	High (*n* = 8)	
Age (years)				0.4076
≤60	7	3	4	
>60	5	1	4	
Gender				0.6788
Male	5	2	3	
Female	7	2	5	
Tumor size				0.0304^∗^
≤2 cm	4	3	1	
>2 cm	8	1	7	

Note: ^∗^*P* < 0.05. ^a^Chi-square test.

## Data Availability

All data generated or analyzed during this study are included in this published article.
